# Some Unpleasant Effects of Cocaine

**Published:** 1888-07

**Authors:** R. O. Cotter

**Affiliations:** Macon, Ga.


### SOME UNPLEASANT EFFECTS OF COCAINE.

By R. O. COTTER, M. D., MACON, GA.

Regardless of the quantity of cocaine literature which has flooded the journals for three or four years, I think the following points in reference to it are well worthy of the attention of those who have to use it. I have used it as an anæsthetic nearly every day for nearly four years past in my practice, and have watched its effects closely. Many eminent authorities seem to think there is no such thing as the cocaine habit, and that the so-called cocaine habitués are simply those unfortunates who have tried to exchange, or add to their opium habit or alcoholism, another stimulant (cocaine). My experience is not worth much upon this point, as it is only a negative one. I have never known personally of a case of cocaine habit, yet I am thoroughly convinced that the continuous use of so powerful a stimulant is obliged to be followed by a correspondingly harmful reaction. For instance, even in the way advocated by Dr. W. A. Hammond, of New

York (*i. e.*), the local application of it to the congested nasal mucous membrane, as in hay fever, it is too powerful a stimulant to use thus *continuously*.

When cocaine was first in use in this country, I allowed myself to occasionally prescribe it incorporated as an ingredient in ointments for the temporary relief of congestions of the nasal mucous membrane. I have not allowed myself to do this for the past two years, however, as I am positive I found that the violent reaction following its use only increased my patient's trouble.

Experience has long since thoroughly convinced me that it acts harmfully in case of conjunctivitis; hence I now never add it to any collyrium. It corrugates, dries and irritates the conjunctiva and cornea, and it does positive harm in ulcers of the cornea if anything like freely used.

I suppose its action here is explained by the reaction following the sudden exsanguination of the circumcorneal tissue. It was at first supposed to be *the* thing to quickly and temporarily dilate the pupil when we wished to make an ophthalmoscopic examination of the fundus of the eye, and also to thus determine the refraction, but I find that its drying and corrugating effect upon the cornea mars the view of the fundus, and also interferes with getting the correct degree of acuity of vision of the patient.

Lately I have used a great deal of it in nasal surgery, in operating upon hypertrophies of the turbinated bones and in sawing off hypertrophies, and ecchondroses of the *septum nasi*. While in New York last winter, I noticed that Dr. F. H. Bosworth, of that city, always used it of the strength of a 20 per cent. solution in this kind of work, and I at first employed it likewise, but my experience soon became so unpleasant that I reduced it to 10 per cent. in my practice. As far as its anæsthetic properties go, I am satisfied with a 10 per cent. solution.

I have had frequent occasion to notice that even in a 4 or 5 per cent. solution, applied in the nostrils once every day or every other day in patients where it was necessary to introduce the Eustachian catheter, where the nostrils were tortuous and the sensibility of the same had to be overcome by cocaine, the secondary effect of the cocaine was quite irritating. In one case of a healthy man, aged 50 years, free of any cancerous, syphilitic or other specific taint, who had a simple hypertrophy of the turbinated bones and septum, I sprayed the nostrils with a 20 per cent. solution of cocaine in order to make an examination previous to operating. Nothing else was used. But in 12 hours, time it had set up such a severe and painful inflammation of the mucous membrane of the vestibule of the nostrils, and which still existed for a month subsequent, that I shall be very happy if the ointment of oxide of lead which I prescribed for my patient shall have cured his nose by the next time he comes to see me. I have, more than once, while using even a 10 per cent. solution, in sawing off ecchondroses of the septum, had my patients exhibit very unpleasant symptoms. These cases are of course exceptional, but I mention them as proof that we must be careful of the free use of the drug. Only yesterday the most serious symptoms I have yet observed from its use were shown. I was removing with the saw, a pretty large hypertrophy from the nasal septum of a young lady. I happen to know that she is a young woman of a great deal of fortitude, and by no means a nervous one. I suppose I had sprayed in the nostril 6 or 7 grains of a 10 per cent. solution of cocaine, and just before I commenced using the saw she began to exhibit very great difficulty in breathing. The epiglottis seemed paralyzed. The larynx was undoubtedly considerably affected, and she evinced such very distressing symptoms of general depression that, although she insisted that I should complete the operation and use more cocaine if necessary, I would not risk removing more than half the hypertrophy at this sitting. I wish to state distinctly that I was not disturbed by the usual symptoms of mental excitement or intoxication which she showed, but it was the alarming depression, and the heart's action was alarmingly accelerated. In fact, I feared that heart spasm would ensue. She was totally unable to walk for two hours, and I was most happy when I saw her symptoms begin to subside. Her intellectual function was not at all affected. Four weeks ago I used the same quantity of cocaine for the same operation in a 10-year-old boy; two hours afterward he had stolen out of the nursery and was at play out doors, having not been affected by the cocaine at all unpleasantly. Cocaine is invaluable in eye and nasal surgery, and I expect to continue to use it, but I shall certainly be more and more careful of its free use. I should think any considerable amount—say over 5 grains, perhaps—would be liable to prove quite dangerous in a case of weak heart, or organic heart disease. I do not know its physiological antidote, but think I would, in case of an overdose of it, give strong coffee, and I also generally advise my patients, if affected unpleasantly by it, to take some good, nourishing soup, well flavored with red pepper—all of which, I am sure, helps to overcome its effects. In cauterizing hypertrophies of the turbinated bones I find that a 5 per cent. solution is sufficient—that is, for chromic acid cautery.

